# Variation in community structure of gall‐inducing insects associated with a tropical plant supports the hypothesis of competition in stressful habitats

**DOI:** 10.1002/ece3.5827

**Published:** 2019-11-22

**Authors:** Letícia F. Ramos, Ricardo R. C. Solar, Henrique T. Santos, Marcilio Fagundes

**Affiliations:** ^1^ Programa de Pós Graduação em Ecologia Manejo e Conservação da Vida Silvestre Universidade Federal de Minas Gerais Belo Horizonte Brazil; ^2^ Programa de Pós Graduação em Biodiversidade e Uso dos Recursos Naturais Universidade Estadual de Montes Claros Montes Claros Brazil; ^3^ Departamento de Genética, Ecologia e Evolução Instituto de Ciências Biológicas Universidade Federal de Minas Gerais Belo Horizonte Brazil

**Keywords:** bottom‐up, community assembly, *Copaiffera langsdorffii*, environmental stress, null models, top‐down

## Abstract

Environmental factors act as drivers of species coexistence or competition. Mesic environments favor the action of parasites and predators on gall communities, while the factors that determine the structure of gall communities in xeric environments remain unknown. We evaluated the structure of gall communities along an environmental gradient defined by intrinsic plant characteristics, soil fertility, and aridity, and investigated the role of competition as a structuring force of gall communities in xeric environments. We created null models to compare observed and simulated patterns of co‐occurrence of galls and used the C‐score index to assess community aggregation or segregation. We used the NES C‐score (standardized C‐score) to compare patterns of co‐occurrence with parameters of environmental quality. Xeric environments had poorer and more arid soils and more sclerophyllous plants than mesic environments, which was reflected in the distribution patterns of gall communities. Values of the C‐score index revealed a segregated distribution of gall morphospecies in xeric environments, but a random distribution in mesic environments. The low availability of resources for oviposition and the high density of gallers in xeric environments reinforce interspecific competition as an important structuring force for gall communities in these environments.

## Graphical abstract

Our assessment of the role of competition in structuring gall communities along a gradient of environmental stress advances the understanding of the role of environmental filters in biotic responses imposed by abiotic changes in a natural environment. Mesic environments favor the action of parasites and predators on gall communities, while competition determines the structure of gall communities in xeric environment.

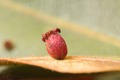

## 1 INTRODUCTION

Historically, interspecific competition is deemed as one of the major forces responsible for shaping the distribution of species in space and time (Connor & Simberloff, 1983; Diamond, 1973). However, competition importance is disputable, especially for phytophagous insects, and some authors argue that food resources were not a limiting factor, and therefore, competition would be absent or very weak (Hairston, Smith, & Slobodkin, 1960; Strong, 1982). Later, with rising knowledge about plant defenses, the role of competition has gained prominence (Kaplan & Denno, 2007; Murdoch, 1966; Reitz & trumble, 2002), and recent studies have highlighted the important role of competition for phytophagous insect community structure (Cornelissen, de Carvalho Guimarães, Rodrigues Viana, & Silva, 2013; Kaplan & Denno, 2007). Indirect mechanisms, such as extreme or unstable environmental conditions, can now be incorporated into models to help understand the role of competition in assembling communities in climate change scenarios.

The structure of gall‐inducing insect communities can be determined by environmental factors (Blanche, 2000; Butterill & Novotny, 2015; Craig, Itami, & Craig, 2007; Cuevas‐Reyes, Quesada, & Oyama, 2006; da Costa, de Siqueira Neves, de Oliveira Silva, & Fagundes, 2011; Price, 2002), by top‐down (Fagundes, Neves, & Fernandes, 2005; Price, 1997) or bottom‐up (Egan & Ott, 2007; Espírito‐Santo, de S. Neves, Andrade‐Neto, & Fernandes, 2007; Hunter & Price, 1992; Malinga, Valtonen, Nyeko, Vesterinen, & Roininen, 2014) mechanisms, and by interactions that occur within the same trophic level, such as interspecific competition (Cornelissen et al., 2013; Fagundes & Fernandes, 2011; Fagundes et al., 2018). Studies have reported that the results of interspecific interactions involving phytophagous insects can be habitat‐dependent (Kuchenbecker & Fagundes, 2018).

The environmental stress hypothesis (ESH) predicts that gall‐inducing insect diversity should be greater in more stressful/xeric habitats (Fernandes & Price, 1988). The mechanisms underlying greater species richness in xeric habitats involve evolutionary and ecological processes (Fernandes & Price, 1988; Price, Fernandes, & Waring, 1987; Ribeiro & Basset, 2007). Among ecological mechanisms, the actions of parasites and predators (third trophic level) are thought to be less effective at regulating gall‐inducing insect diversity in xeric habitats, thus leading to greater diversity of gallers in such habitats (Castellanos, Cuevas‐Reyes, Rios‐Casanova, Oyama, & Quesada, 2006). Studies have also shown that plants growing on water‐ and nutrient‐deprived soils are more sclerophyllous (Poorter, Niinemets, Poorter, Wright, & Villar, 2009) and accumulate higher levels of chemical compounds (Fagundes et al., 2018). Thus, internal‐feeding gall‐inducing insects attacking such plants would experience greater protection from their natural enemies (Hardy & Cook, 2010; Ribeiro & Basset, 2007). These hypotheses have been supported by several studies conducted in tropical (Fernandes, Gonçalves‐Alvim, & Carneiro, 2005; Jesus, Silva, & Fernandes, 2012; Julião, Almada, & Fernandes, 2014; Lara, Fernandes, & Gonçalves‐Alvim, 2002) and temperate (Fernandes, Duarte, & Lüttge, 2003; Fernandes & Price, 1988, 1992) habitats.

Recent studies have shown that interspecific competition be an important phenomenon capable of shaping the community structure of sedentary organisms, such as gall‐inducing insects (e.g., Cornelissen et al., 2013; Fagundes et al., 2018). Some characteristics of the interaction between galling insects and their host plants make this system suitable for better understanding the processes that structure natural communities. For example, gall‐inducing insects have high specificity with regard to their host plant and the target organ to oviposition (Carneiro et al., 2009; Joy & Crespi, 2007) they synchronize the oviposition period with the plant phenological period, where there is growth but the tissues are not yet lignified (Whitham, 1978; Yukawa, 2000). Soon after oviposition, hatched larvae of galling insects induce rapid morphological and physiological changes in host plant tissue, draining resources for their own development (Höglund, 2014; Ollerstam, Rohfritsch, Höglund, & Larsson, 2002), which potentially interferes with the oviposition behavior of females of other gall‐inducing insects species (Cornelissen et al., 2013). Because they are sessile and highly specific, galls are excellent models for understanding the effects of interspecific competition on community structuring (Cornelissen et al., 2013; Fagundes et al., 2018).

Interspecific competition in endophagous insect communities is affected by the direct action of bottom‐up and top‐down forces, which put pressure on the community (Kaplan & Denno, 2007). Because gall‐inducing insects have greater richness and abundance in habitats under higher stress and lower incidence of natural enemies (Castellanos et al., 2006; Fernandes & Price, 1988), competition can be expected to exert pressure in this community. One way to test these competitive hypotheses in natural communities when the manipulation of species is not possible is through the use of null models (e.g., Ribas & Schoereder, 2002). Null models are models of communities that have characteristics of their real equivalents, while the randomness of the distribution of the species is maintained, specifically excluding the effects of biological interactions (Gotelli & Graves, 1996).

Adverse environmental characteristics, such as extreme temperatures, low water availability, high light incidence, and low soil fertility, are stress factors that directly affect plant development (Lázaro‐Nogal et al., 2015; Pennington & Collins, 2007; Sapijanskas, Paquette, Potvin, Kunert, & Loreau, 2014). Variation in leaf characteristics and tree growth have been correlated with the efficiency of resource use and with plant phenotypic plasticity in response to such abiotic variation (Chaturvedi, Raghubanshi, & Singh, 2014; Lázaro‐Nogal et al., 2015). For example, leaf sclerophylly is a plant response to stress factors, where leaves with thicker cuticles and greater stomatal density minimize water loss through transpiration (Bussotti, Pollastrini, Holland, & Brüggemann, 2015; Reich et al., 2003; Williams & Black, 1993). These morphological and physiological adaptations of plants can determine the colonization and survival of plant species in different environments (Sultan, 2001). Therefore, measuring characteristics of plants can be a suitable surrogate for the combined effect of stress factors acting on each individual. In this sense, species with wide geographical distributions must undergo changes in their development according to the great variation in the conditions that they are exposed to, with the effect escalating to higher trophic levels (Souza et al., 2018).

*Copaifera langsdorffii* (Fabaceae) has a wide geographical distribution and occurs in varied environments that vary widely in rainfall and soil quality. The species hosts the greatest richness of gall‐inducing insects in the Neotropical Region, which change as a function of habitat and host quality (Costa, 2016). Using this system, we seek to understand how interactions in communities can be modified under various environmental conditions, and how possible climate change can affect the structure of communities. We hypothesized that interspecific competition would be prevalent in xeric environments. We predicted that plants should possess more sclerophyllous leaflets and greater richness and abundance of gall‐inducing insects in xeric environments.

## 2 MATERIALS AND METHODS

### 2.1 Study system

*Copaifera langsdorffii* Desf. (Fabaceae: Caesalpinioideae) is a tropical arboreal plant species that can reach between 8 and 25 m in height. The species has a wide geographical distribution, occurring in Argentina, Bolivia, and throughout the Brazilian cerrado, Atlantic Forest, and Amazon (Almeida, Poença, Sano, & Ribeiro, 1998; Costa et al., 2016). The species exhibits a high degree of phenotypic plasticity in seed size and other morphological and physiological traits, which explain the vast geographical distribution of this species (Souza & Fagundes, 2014). This plant has a remarkable period of deciduousness from July to August, with new leaves growing soon thereafter (Souza, Solar, & Fagundes, 2015). Thus far, *C. langsdorffii* has been recorded to host 24 gall‐inducing insect species (Costa, Fagundes, & Neves, 2010; Fagundes, 2014). The wide geographical distribution of the species and the architecture of its plants are factors that probably favored diversification of the gall‐inducing insects that this species hosts (da Costa et al., 2011; Fagundes, 2014).

### 2.2 Study areas

**Figure 1:**
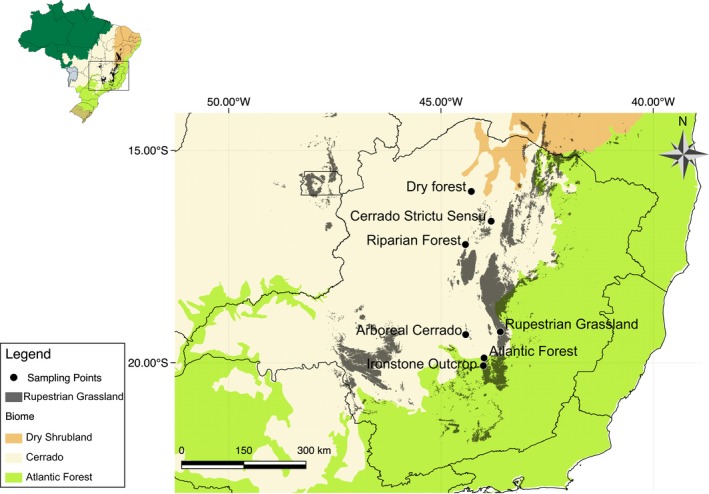
Map of the seven sites/populations in the state of Minas Gerais, Brazil

This study was developed at seven sites with the presence of a population of *C. langsdorfii*, each located in a different plant formation (Figure 1, Table 1). The sites were chosen to include a gradient of environmental stress, ranging from dry environments, such as rupestrian grasslands and ironstone outcrops, to humid environments, such as Atlantic forest and riparian forest. The distance between sampling areas ranges from 90 to 500 km.

**Table 1:** Historical climatic characterization of the environmental variables of the study sites. Mean maximum temperature (°C) (T.max.), mean minimum temperature (°C) (T.min) and Piche evaporation (mm) (Evapo). Data for previous 55 years, obtained from INMET website, August 2015. The data obtained from meteorological stations closest to the study sites

Habitat	Coordinates	Elevation	Insolation	T‐max	T‐mim	Evapo
Rupestrian grasslands	20°04′S, 43°59′W	1,423	185.21	28.12	14.65	89.43
Ironstone outcrops	19°16′S, 43°35′W	1,200	168.73	28.05	15.39	87.71
Cerrado sensu* stricto*	16^o^40′S, 43^o^48′W	652	222.86	29.73	17.54	138.52
Arboreal cerrado	19°20′S 44°24′W	732	221.39	28.51	15.89	88.66
Dry forest	15º58′S, 44º16′W	826	240.36	31.44	18.39	143.51
Atlantic forest	19°53'S 43°58'W	915	208.05	27.13	17.26	116.92
Riparian forest	56°25′S, 80°96′W	480	229.28	31.03	18.30	125.62

The rupestrian grasslands were evaluated in a conservation unit of Serra do Cipó State Park (area of ca. 33,000 ha). This environment is dominated by tree–shrub species with sclerophyllous leaves, and shallow and sandy nutritionally poor soils (Giulietti,Menezes, Pirani, Meguro, & Wanderley, 1987). The Ironstone outcrops are located in a conservation area in the Calçada mountain range of approximately 1,100 ha. These environments have nutrient‐poor hight iron content soils, and the plants are sclerophyllous adapted to the sudden variations in temperatures and high winds (Giulietti et al., 1987; Oliveira‐Filho & Ratter, 2002). The cerrado sensu stricto is located in a private reserve of 20 ha, the soils are acidic and poor in nutrients, and the vegetation has leathery leaves, generally taken as an adaption to dry conditions (Oliveira, 1998; Rizzini, 1997). The arboreal cerrado is located in a National Forest with 203.29 ha, the soils are deep and slightly acidic with medium content of organic matter from the fall of the leaves in the dry season, and the vegetation is large with closed crowns (Rizzini, 1997; Sano, 2008). The deciduous forests (dry forests) were evaluated in a private reserve with 350 ha, the vegetation is large (which can exceed 25 m), and the total leaf abscission in the dry season is the main feature of this environment. The soils are alkaline and rich in nutrients, and leaf fall contributes to soil fertility (Fagundes & Fernandes, 2011; Sano, 2008). The Atlantic forest was evaluated in a remnant of forest in the UFMG ecological station with an area of ca.114 ha. The vegetation has a large size forming a continuous canopy and high humidity. Soils are moist, shallow and poor in mineral nutrients; however, decomposition of organic matter is an important factor in nutrient availability for plants (Sano, 2008). The riparian forest was evaluated in an environmental protection reserve with an average area of ca. 98 ha. The vegetation forms corridors following the riverbed with, the fertile, the soils and high relative humidity (Oliveira‐Filho & Ratter, 2002).

### 2.3 Measurements of environmental stress

We obtained meteorological data for each of the studied sites for the 24 months preceding the study—a period corresponding to vegetative investment by each plant (Fagundes et al., 2016). The data were obtained from the closest meteorological station to each of the sites using the public platform of the Brazilian Meteorological Institute (http://www.inmet.gov.br). Using the meteorological data, we calculated the aridity index (AI) for each site using the formula: AI = (*P*/PET), where *P* is total monthly precipitation, and PET is potential monthly evapotranspiration (Picotte, Rhode, & Cruzan, 2009). In this case, lower AI values indicate higher aridity (i.e., a more water‐deprived habitat).

We also measured the chemical characteristics of the soil of each of the studied sites. We collected soil samples (three samples at 10 cm depth) below each individual plant of *C. langsdorffii* selected for the study (45 soil samples at each site). The soil samples of each site were homogenized to obtain a single composite soil sample per site, which were then submitted to chemical analysis at the Soil Laboratory of the Federal University of Minas Gerais.

### 2.4 Biological data collection

Biological data were collected in the months of April and May of 2015, before leaf fall, but when galls are completely established in the plants (Fagundes, 2014). Fifteen healthy and adult individuals (i.e., individuals who have undergone a reproductive phase) of *C. langsdorffii* were selected at each sampling site. The plants have irregular distribution in the environment but while assuring a minimum distance of 30m between individuals in order to maximize independence among samples. Ten terminal branches were removed from the crown of each plant (Costa et al., 2010), packed in plastic bags, and taken to the laboratory. The last three leaves of the last vegetative investment (annual plant growth easily identified by the visible scar on the branch) were selected from each branch for measuring other parameters.

The second pair of leaflets of each of the 10 branches removed per plant (300 leaflets for each site) was used to determinate leaf size and specific foliar mass (sfm) of *C. langsdorffii*. A disk (0.38 cm^2^) was removed from each leaflet, dried in oven at 40°C for 92 hr, and individually weighed in an analytical balance (Cornelissen et al., 2003; Dwyer, Hobbs, & Mayfield, 2014). The size and mass of the leaf are proxy for foliar sclerophylly of the plants, so in this work we use only the sfm in the analyses, but the size of the leaf follows the same pattern. A total of 137 leaflets were evaluated in each population to determine the distribution pattern of galls. The method selected required that all leaflets possess at least one species of gall. In this way, it was possible to create a presence/absence matrix of galls per leaflet, where presence is equivalent to more than one species on a leaflet, and absence is equivalent to a single species of gall on the leaflet. Such a matrix was created separately for each population/site evaluated. Sampling was standardized at 137 leaflets per each population because this was the maximum number of leaflets found with galls in all populations/sites. The galls present on each leaflet were counted and identified according to their morphology, color, texture, and size (da Costa et al., 2011; Fagundes, 2014).

### 2.5 Data analysis

We performed a principal component analysis (PCA) to obtain a summary of the soil variables by ordinating soil traits of the seven sites using soil fertility characteristics (Ph, H^+^ Al, Al3^+^, Ca, Mg, P, K, SB, t, m, V). We used generalized linear models (GLMs) followed by deviation analysis (ANODEV) to evaluate whether slm varied as a function of sites and/or soil fertility. The identity of each site and the scores of the first axis of the PCA were used as explanatory variables, while slm was used as the response variable. Models were evaluated using the “F” test with Gaussian error distribution.

Variation in gall‐inducing insect richness and abundance among sites was also tested using GLMs followed by ANODEV. In this case, gall‐inducing insect richness, or abundance, was the response variable and sites/population was the explanatory variable. The error distribution used was Poisson, corrected by quasi‐Poisson as necessary. Finally, to test the effects of slm on gall‐inducing insect richness and abundance we used generalized linear mixed‐effects models (GLMEr; Bolker et al., 2009). Here, gall‐inducing insect richness, or abundance, was the responses variable and slm was the explanatory variable, while site/population was assumed to be a random effect, based on the Poisson distribution. The models were tested by comparison with a null model using a chi‐square test.

### 2.6 Analysis of co‐occurrence and community structure

We used null models to compare observed and simulated patterns of the occurrence of gall‐inducing insects within each population/site. Data on gall species of the leaflets were transformed into presence/absence matrices in which columns represented individual leaflets, while rows represented species of galls. An individual matrix was created for each site/population, which were analyzed separately. The null hypothesis predicts that the presence of one gall species on a leaflet does not influence the presence of another on the same leaflet. Gall occurrence was analyzed using the EcoSim software (Gotelli & Entsminger, 2001). Thus, we used the *C‐score* index (Stone & Roberts, 1990) as the metric quantifying co‐occurrence patterns, or “checkerboard units” (CU) between possible pairs of galls species, given by the formula: CU = (*ri* − *S*) (*rj* − *S*). The *C‐score* measures the average frequency of all possible pairs of species interacting at least once in the matrix. We used a “fixed‐fixed” algorithm with 5,000 randomizations, where the rows and columns of the original matrix were preserved (Gotelli & Entsminger, 2001).

Nine algorithms have been described for testing null models, with the most appropriate choice depending on the structure of the original matrix (Gotelli, 2000). The *C‐score* indices (Stone & Roberts, 1990) measure the pattern of exclusion of species, which reflects competitive interactions. Thus, this index is an ideal model for work of this type where it is not possible to test the effect of the removal of one gall versus the removal of others (Cornelissen et al., 2013). Nonetheless, this index does not incorporate species abundance (Gotelli & Graves, 1996); on the other hand, it is less prone to type I and type II errors (Gotelli, 2000). Some authors point out that analyzing the distribution of species with the *C‐score* reveals patterns of segregation when species do not interact simply because they use different resources (Diamond & Gilpin, 1982; Gotelli & Rohde, 2002). In this work, the evaluated species use the same resource (leaflet), and so the index is suitably applicable.

We obtained a single index value for each site/environment, which reflects the pattern of structure of the local community. However, the normalized effect size [(NES *C‐score* = observed‐expected scores)/expected scores) (Ulrich, Jabot, & Gotelli, 2016)] was used so that the indexes were comparable among site/environment. High values of the NES *C‐score* index indicate low co‐occurrence of species in the community (Gotelli, 2000).

To test the hypothesis that competition operates in xeric environments, we built models in which the “NES *C‐score*” of each population/site was tested against the indicators of environmental (soil fertility and aridity index) and plant (slm) stress. For this, we used GLMs, where mean slm (of the plants of each population/site) was used as the explanatory variable and NES *C‐score* as the response variable, with the Gaussian distribution.

All models described above were submitted to residual analysis to verify their adequacy and adjustment to the chosen error distribution (Crawley, 2007). All GLMs, ANOSIM (Vegan package) (Jari Oksanen et al., 2019), and generalized linear mixed‐effects models (GLMEr) (Bates, Mächler, Bolker, & Walker, 2014) were performed in R software (R Core Team, 2017).

## 3 RESULTS

**Figure 2:**
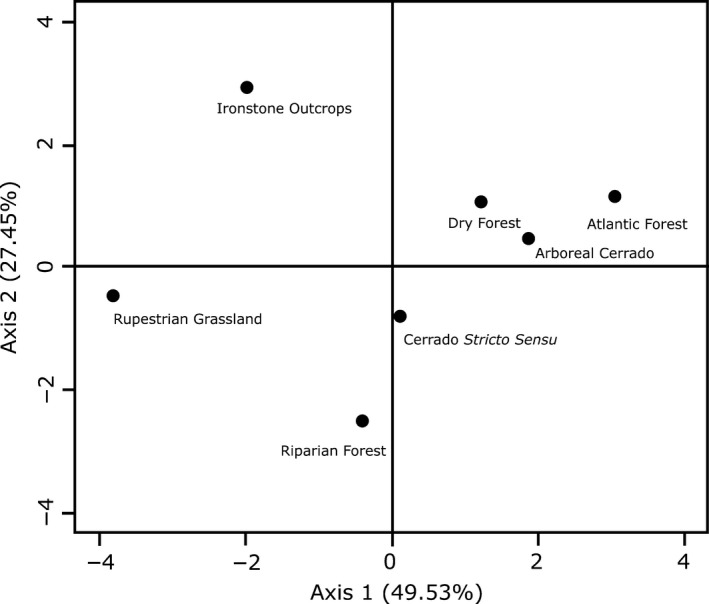
Principal component analysis (PCA) of soil quality indicator parameters of the seven study sites

The first two axes of the PCA together explained 76.98% of the total variation in soil quality among the seven sites (Figure 2). Sites with low soil quality (rupestrian grasslands and ironstone outcrops) were negatively related to the first axis, while sites with higher soil quality (arboreal cerrado, dry forest, and Atlantic forest) were positively related to the first axis. In addition, sites with average soil quality exhibited a weak relationship with the first axis of PCA. Therefore, the first axis of PCA represents a gradient of soil quality among the study sites.

**Figure 3:**
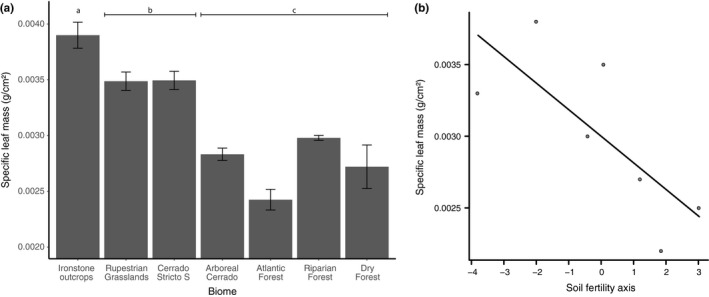
(a) Variation in specific leaf mass of plants of *Copaifera langsdorffii* among the seven study sites/environments with different levels of stress. Same letters on bars represent grouping by contrast analysis. (b) Variation of specific leaf mass by soil fertility (axis 1 of PCA)

The slm of plants of *C. langsdorffii* varied among sites/environments (*F*_1,6_ = 26.507 *p* < .001). Plants grown in more stressful sites with poorer soils (e.g., rupestrian grasslands, ironstone outcrops, and cerrado sensu* stricto*) have more sclerophyllous leaves than plants growth in more humid sites (e.g., arboreal cerrado, dry forest, riparian forest, and Atlantic forest; Figure 3a). More importantly, we found a positive relationship between soil quality (here represented by scores of the first axis of the PCA) and leaf sclerophylly (i.e., slm) (*F*_1,6_ = 6.70, *p* = .04; Figure 3b).

**Figure 4:**
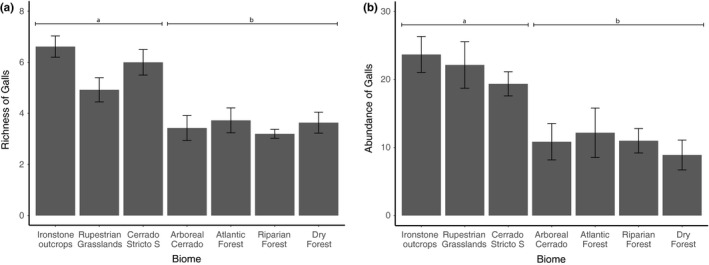
(a) Variation in gall richness and (b) abundance on plants of *Copaifera langsdorffii* among the seven studied sites/environments with different levels of stress. Same letters on bars represent grouping by contrast analysis

Richness (deviance = 28.915, *F* = 9.325, *p* < .001) and abundance (deviance = 176.55, *F* = 4.656, *p* < .001) of gall‐inducing species per plant varied among the study sites, with more stressed sites having greater richness (Figure 4a) and abundance (Figure 4b) than more mesic sites. However, slm of plants did not affect the richness (deviance: 325.83, *χ* ^2^ = 0.24, *p* = .101) or abundance (deviance: 648.8, *χ* ^2^ = 234.4, *p* = .276) of gall‐inducing insects.

The patterns of co‐occurrence of gall‐inducing species also varied among sites (Table 2). In fact, the null models showed that the communities of gall‐inducing insects at the xeric sites were not randomly distributed, with greater co‐occurrence than expected by chance, indicating that biotic forces are structuring these communities of herbivores. On the other hand, the observed pattern of gall‐inducing insect co‐occurrence did not differ from that expected by chance in mesic environments. In this case, biological interactions cannot be used to explain the distribution of gall‐inducing insects on plants of *C. langsdorffii*.

**Table 2:** C‐score indices of the occurrence of galls species on Copaifera langsdorffii in the seven populations/sites analyzed. Maximum and minimum indices were calculated from 5,000 randomizations of the original matrix. The *p*‐values were obtained by the bi‐flow test and represent the probability that the observed index is greater, less than, or equal to that expected by randomized matrices. Observed (obs), expected (exp)

Habitat	Indices for randomized matrices		Observed index	*p*‐Values	
	Minimum	Maximum		Obs. ≥ exp.	Obs. ≤ exp.
Rupestrian grasslands	120.30	123.84	123.25	**.001**	.98
Ironstone outcrops	74.83	76.85	76.81	**.0002**	.9998
Cerrado sensu* stricto*	114.82	121.92	121.66	**.003**	.9970
Arboreal cerrado	128.66	132.98	130.78	.552	.456
Dry forest	115.62	118.62	117.89	.252	.757
Riparian forest	99.84	103.24	101.92	.15	.846
Atlantic forest	122.45	127.21	123.40	.89	.11

**Figure 5:**
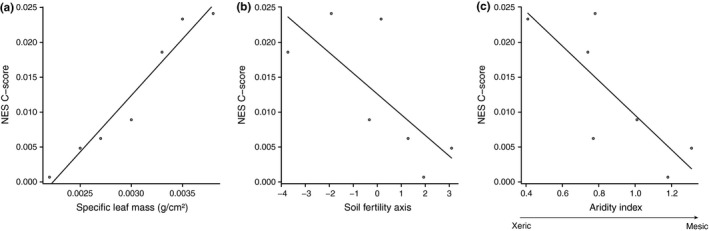
Relationship between standardized NES C‐score values for all evaluated sites and (a) specific leaf mass, (b) soil fertility (axis 1 of the PCA), and (c) aridity index (obtained with environmental variables of each site)

Finally, we observed that mean leaf sclerophylly of host plants and environmental stress affected the observed values of the *C‐score* index of gall‐inducing insects (Table 3). In fact, leaf sclerophylly (Figure 5a) and soil quality (Figure 5b) were positively related to the *C‐score* index of the gall‐inducing insect communities, while the aridity index was negatively related (Figure 5c).

**Table 3:** Deviance analysis of the appropriate minimum models to evaluate the effects of stress indicators (environmental and plant) on the co‐occurrence of galls of *Copaifera langsdorffii* in the seven different study sites/populations

Response variables	Explanatory variables	Deviance	Residual deviance	DF	*F*	*p*
NES *C‐score*	Specific leaf mass	0.005	0.0054	6	94.7	<.01
NES *C‐score*	Aridity index	0.001	0.0014	6	10.5	.04
NES *C‐score*	Soil fertility (PC1)	0.0001	0.0002	5	8.42	.04

## 4 DISCUSSION

Our assessment of the role of competition in structuring gall communities along a gradient of environmental stress advances the understanding of the role of environmental filters in biotic responses imposed by abiotic changes in a natural environment. Co‐occurrence analysis and the use of null models (Stone & Roberts, 1990) allowed us to conclude that there is less co‐occurrence of galls on the same leaflet in xeric environments, whereas species co‐occur freely on the same leaflet in mesic environments. With this, we can affirm that competition acts in the structuring of gall communities, but only in xeric environments. We also found structural changes in plant characteristics (leaf sclerophylly) and in the pattern of occurrence of galls that were dependent on the environments where they were found, with greater richness, abundance and aggregate patterns in xeric environments. Our results are very similar to patterns of gall richness and abundance previously described in the literature (Fernandes et al., 2005; Lara et al., 2002). This set of results allows a better understanding of how environmental changes (e.g., temperature increase, desertification) can modify ecological interactions and the resultant impacts on biodiversity.

### 4.1 Plastic responses of plants to environmental variation

Variation in the structural traits of plants along environmental stress gradients can be seen as adaptive strategies, which ultimately determine the range of conditions and resources in which species can successfully survive and reproduce (Sultan, 2001). The environments studied here represent a gradient of hydric stress, which is manifested in structural differences of leaves in the different habitats, so that changes in these characteristics can be used as indicators of environmental stress. Hence, the changes we observed here in vegetative parameters of *C. langsdorffii* can alter the interactions between galling insects and their host plants, and may even have effects scaling to higher trophic levels (Craig et al., 2007; Egan & Ott, 2007).

### 4.2 Communities of galling insects

Many studies have shown that the distribution and survival of galling insect communities are dependent on the characteristics of the environment where the host plant is established, and which ultimately shape such traits of galling insects (Blanche, 2000; Fernandes & Price, 1992; Price, 2002). Stiling and Moon (2005) showed that the quality and availability of plant resources are the bottom‐up factors that most affect the growth, establishment, survival, and feeding preference of herbivores. Furthermore, host plant quality can directly influence the third trophic level (natural enemies) (Legrand & Barbosa, 2003) and favor a high diversity of galls in the xeric environments (Fernandes & Price, 1992). We found a pattern consistent with those described in the literature, that is, greater richness and abundance of galling insects in the more xeric environments (Fernandes et al., 2005; Fernandes & Price, 1992; Jesus et al., 2012; Julião et al., 2014; Lara et al., 2002). The plants inhabiting xeric environments tend to have higher availability of amino acids and free organic nitrogen compounds, which means that they are more nutritious (Price, 1991; White, 1969). These plants also have a limited capacity to present induced defense mechanisms (Fernandes, 1990, 2000; Fernandes & Negreiros, 2001; Hoglund, Larsson, & Wingsle, 2005), and thus favor a greater amount of galling insects (Barbosa & Fernandes, 2014). Therefore, it is plausible that there will be a higher abundance and richness of specialized galling insects in these xeric environments, as previously postulated by Fernandes and Price (1992).

### 4.3 Gall community structure

We showed that habitat quality affects the diversity of gall‐inducing insect communities and that the structure of these communities varies across the range of stress. Some studies have shown competition between herbivorous insects to be a fairly common (Cornelissen et al., 2013; Kaplan & Denno, 2007; Reitz & Trumble, 2002) and central process to community structure (Denno, McClure, & Ott, 1995; Jennings, Krupa, Raffel, & Rohr, 2010; Kaplan & Denno, 2007; Reitz & Trumble, 2002). We also showed that the distribution patterns of galls species differed among the studied environments, with more xeric environments having a more segregated community pattern. Considering that we also found that individuals inhabiting xeric environments have smaller leaflets, this pattern is likely to be result of competition for oviposition sites. As expected, galling species are distributed randomly among leaflets in mesic environments, with no patterns of segregation.

Several factors may explain the nonrandom distribution of galling insect species in xeric environments, such as the high abundance of galls (Fernandes & Price, 1992; Price et al., 1987), the lower availability of resources (space dispute, suitable places for oviposition), and less activity of natural enemies, all of which would allow more effective infection by galling insects (Fernandes et al., 2005; Jesus et al., 2012; Lara et al., 2002). Another factor that operates on the scale of the leaflet, and which potentially favors the segregation of gall species, is interference competition. Organisms that have a sessile habit are more susceptible to competitive influences because once they are established they cannot escape from their neighbors, with the outcome of such an interaction resulting in a segregated distribution pattern (Cornelissen et al., 2013; Kuebbing, Souza, & Sanders, 2014; Sanders, Gotelli, Heller, & Gordon, 2003). For example, using invasive plants as a model (i.e., sessile organisms), Sanders et al. (2003) demonstrated that interference competition was responsible for the segregation pattern they found, which was similar to that found by the present study. Although galling insects occur on small islands of resources (e.g., the leaflet), we observed that in environments where leaflets are smaller (xeric environments), only a single gall morphospecies occurs on any given leaflet; that is, space might be moderating the choice of oviposition site. Similar results have been found for other systems, such as the invasive plants mentioned above (Sanders et al., 2003), arthropods (Ellwood, Manica, & Foster, 2009), and galls (Cornelissen et al., 2013), and in these cases, there was also evidence that competition was the main process responsible for community structure.

We used null models to evaluate the effect of competition on gall communities in different environments since they were the most viable (Cornelissen et al., 2013; Ribas & Schoereder, 2002). The alternative of conducting exclusion experiments was not feasible since the effects of the removal of one gall on a competitor are difficult to detect (Cornelissen et al., 2013). The significant relationship between NES *C‐score* indices and the indicators of environmental stress (specific leaf mass, soil fertility, and dryness index) found here allows us to conclude that bottom‐up forces, such as competition for foliar resources, are important drivers of gall community structure. Xeric environments tend to have high NES *C‐score* values, indicating a low co‐occurrence of the species evaluated in the study sites and revealing a segregated pattern of gall species in habitats where more sclerophyllous traits prevail.

In conclusion, we showed how environmental variation can change plant structures and influence higher trophic levels (i.e., galling insects). Xeric environments have nutrient‐poor and arid soils with plants that are more sclerophyllous (high foliar mass), yet these environments have high richness and abundance of gall‐inducing insects. Likewise, the co‐occurrence of species also responded to environmental variation, with less co‐occurrence of galls in xeric environments and no distribution pattern in mesic environments. The distribution of species among different environments is determined by assembly rules, mainly interspecific competition (Diamond, 1986), which is defined by the availability of resources. This work represents a step toward a better understanding of the evaluation of abiotic effects in tropical environments and shows how species interactions can be affected by unstable environmental conditions. In this way, we accept our initial hypothesis and argue that competition is an important force in structuring the gall communities in xeric environments.

## CONFLICT OF INTEREST

There are no conflicts of interest among authors.

## AUTHOR CONTRIBUTIONS

Ramos L, Fagundes M, and Solar RR contributed to the experimental design, performed data analysis, and wrote the article. Santos H and Ramos L collected the samples and created the database. All authors contributed to editing a late version of the article.

## Data Availability

All data used in the production of this article are available via Dryad: https://doi.org/10.5061/dryad.nzs7h44mp
